# Integrated analysis identifies a pathway-related competing endogenous RNA network in the progression of pancreatic cancer

**DOI:** 10.1186/s12885-020-07470-4

**Published:** 2020-10-02

**Authors:** Fuqiang Zu, Peng Liu, Huaitao Wang, Ting Zhu, Jian Sun, Weiwei Sheng, Xiaodong Tan

**Affiliations:** 1 Shengjing Hospital of China Medical University, Shenyang, 110004 Liaoning China; 2 Department of General Surgery, Shengjing Hospital of China Medical University, Shenyang, 110004 Liaoning China; 3 Department of Gastrointestinal Surgery, the First Hospital, China Medical University, Shenyang, 110001 Liaoning China

**Keywords:** Integrated analysis, Cancer pathways, Competing endogenous RNA, Pancreatic cancer, Progression

## Abstract

**Background:**

It is well acknowledged that cancer-related pathways play pivotal roles in the progression of pancreatic cancer (PC). Employing Integrated analysis, we aim to identify the pathway-related ceRNA network associated with PC progression.

**Methods:**

We divided eight GEO datasets into three groups according to their platform, and combined TCGA and GTEx databases as a group. Additionally, we screened out the differentially expressed genes (DEGs) and performed functional enrichment analysis in each group, and recognized the top hub genes in the most enriched pathway. Furthermore, the upstream of miRNAs and lncRNAs were predicted and validated according to their expression and prognostic roles. Finally, the co-expression analysis was applied to identify a pathway-related ceRNA network in the progression of PC.

**Results:**

A total of 51 significant pathways that common enriched in all groups were spotted. Enrichment analysis indicated that pathway in cancer was greatly linked with tumor formation and progression. Next, the top 20 hug genes in this pathway were recognized, and stepwise prediction and validation from mRNA to lncRNA, including 11 hub genes, 4 key miRNAs, and 2 key lncRNAs, were applied to identify a meaningful ceRNA network according to ceRNA rules. Ultimately, we identified the PVT1/miR-20b/CCND1 axis as a promising pathway-related ceRNA axis in the progression of PC.

**Conclusion:**

Overall, we elucidate the pathway-related ceRNA regulatory network of PVT1/miR-20b/CCND1 in the progression of PC, which can be considered as therapeutic targets and encouraging prognostic biomarkers for PC.

## Background

Despite advances in treatments for pancreatic cancer (PC), the outcome of patients remains unsatisfying, with a five-year survival of < 5% [1]. What’s worse, owing to a lack of specific symptoms in the early stage, the majority of patients with PC are in advanced stages, which lost the chances for a radical resection [2]. Therefore, it’s urgently necessary to figure out how PC occurs and progresses, and recognize novel therapeutic targets for PC.

As is well known that cancer-related pathways play significant roles in the progression of PC, and genes with similar functions cluster together to form a regulatory pathway [3]. what’s more, non-coding RNAs (ncRNAs) are found to be abundant in the human genome, which can interact with coding RNAs. Although ncRNAs can’t directly code functional protein, accumulating evidence has indicated that ncRNAs, including miRNA, long noncoding RNA (lncRNA), and circular RNA (circRNA), acts as a vital part in oncogenesis and tumor progression of various cancers [4–6]. Moreover, multiple RNAs can interact with each other via miRNA response elements (MREs) and assemble as a competing endogenous RNA (ceRNA) network [7]. In this network, lncRNA can act as “sponges” to absorb and bind miRNA, thereby weakening their binding ability to mRNA and regulating gene expression at the transcriptional and post-transcriptional levels. Notably, emerging data have backed that the ceRNA network might play a pivotal role in cancer progression and metastasis, including breast cancer, ovarian cancer, as well as PC [8–10]. Therefore, it is necessary to figure out the relationship between the ceRNA network and cancer-related pathways using integrated bioinformatics analysis.

In this work, we collected eight gene expression omnibus (GEO) datasets and divided them into three groups according to their platform. Moreover, we combined the cancer genome atlas (TCGA) and the genotype-tissue expression (GTEx) databases as a group because of the limitation of a few normal samples in TCGA databases. Additionally, we screened out differentially expressed genes (DEGs) and performed KEGG enrichment analysis in each group, and looked for all the genes in the most enriched KEGG pathway. Subsequently, protein-protein interaction (PPI) networks were constructed by a string database [11], and the top 20 hub genes were recognized through Cytoscape software [12]. Taken the ceRNA hypothesis into account, lncRNA can diminish miRNA activity via adsorptive action, thereby the qualified candidate lncRNA should be negatively connected with miRNA expression and positively related to the mRNA level at the same time [7]. Therefore, we predicted gene-related upstream miRNA via the miRTarBase database [13] and miRNA-linked upstream lncRNA through the miRNet dataset [14] following this hypothesis. Ultimately, a novel pathway-related ceRNA regulatory network in the progression of PC was successfully identified. Generally, a better understanding of the pathway-related ceRNA network can shed light on the origin of PC, and established potential diagnostic biomarkers and therapeutic targets.

## Methods

### Data selection

To avoid biases caused by single or small numbers of cohorts, we performed a systematic retrospective analysis by screening all the available microarray datasets in the GEO database (www.ncbi.nlm.nih.gov/geo/). Inclusion criteria are as follows: (1) human pancreatic tissue samples; (2) including tumor and non-tumor samples; (3) > 10 samples. Eventually, eight GEO datasets were selected and divided into three groups according to their platform, including GPL570 ([HG-U133_Plus_2] Affymetrix Human Genome U133 Plus 2.0 Array), GPL6244 ([HuGene-1_0-st] Affymetrix Human Gene 1.0 ST Array), and GPL13667 ([HG-U219] Affymetrix Human Genome U219 Array). To increase the reliability of our research, we combined the cancer genome atlas (TCGA) and the genotype-tissue expression (GTEx) databases as a group because of the limitation of few normal samples in TCGA databases.

### Identification of DEGs and functional annotation analysis

RNA-seq raw data of TCGA and GTEx datasets were obtained from the UCSC website totally including 178 cancer samples and 171 normal tissues (https://xena.ucsc.edu/public/) [15–17]. The FPKM (fragments per kilobase of transcript per million mapped reads) data from GTEx were log2(x + 0.001) transformed, and the data from TCGA were log2(x + 1) transformed. To Increase comparability, both forms were unified as log2(x + 1) and normalized through the normalizeBetweenArray function of the “LIMMA” package from R software (version 3.6.1) [18]. Also, the raw data from GEO datasets were obtained and standardized using “RMA” methods [19]. Stepwise, “LIMMA” package was performed to screen differentially expressed genes (DEGs) in each group with thresholds of |log2FC| > 1, adjust *P* value < 0.05. Subsequently, to elucidate the potential functions of those DEGs, we performed the gene ontology (GO) functional enrichment analysis and KEGG pathway analysis via DAVID v6.8 software (https://david.ncifcrf.gov/) [20], and Enrichr (http://amp.pharm.mssm.edu/Enrichr/) [21]. The top 10 enriched GO terms and KEGG pathways were visualized with cut-off criteria of adjust *P* value < 0.05 [22]. Then, the most enriched pathway-related genes were identified for subsequent analysis.

### Pathway-related hub genes identification and validation

The STRING v11.0 database (https://string-db.org/) was a widely-used searching tool for known protein interactions, and was conducted to reveal a PPI network for pathway-related genes with a combined confidence score ≥ 0.4. Next, we utilized CytoHubba, an app in Cytoscape software (Version 3.7.2), which could explore key nodes and fragile motifs in the PPI network, to identify the top 20 hub genes according to their connection degree. Subsequently, we validated the expression levels of hub genes using GEPIA databases that contain 408 PC samples and 211 normal controls from TCGA and Genotype-Tissue Expression GTEx data (http://gepia.cancer-pku.cn/index.html) [23]. And the threshold value was set as |logFC| > 1 and *p* < 0.01. Ultimately, the prognostic roles of key genes were evaluated through the Kaplan-Meier plotter (http://kmplot.com) [24], which includes 177 samples from PC. The hazard ratio with 95% confidence interval and log-rank *P*-value were also automatically worked out and presented on the Web page. And *P* < 0.05 was viewed as a statistical difference.

### Prediction of upstream miRNA and lncRNA and correlation analysis

To obtain comprehensive and reliable prediction results, miRTarBase database that included the experimentally validated microRNA-target interactions was applied to predict the upstream miRNAs of hub genes. Only these miRNAs that proved by strong evidence, including reporter assay, western blot, and qPCR methods were considered as candidate miRNAs. Next, miRNA-Seq data were obtained from TCGA databases to evaluate the expression role of candidate miRNAs. Furthermore, the miRNet database is a user-friendly tool that integrates platform linking miRNAs, targets, and functions, which was employed to find out potential lncRNAs binding to validation miRNAs (https://www.mirnet.ca/miRNet/). Also, the GEPIA database was applied to assess the expression role of lncRNA. KM plot databases were performed to verify the survival effect of potential miRNA and lncRNAs. And we validated the survival outcome of miRNAs in the OncoLnc database to increase the reliability of our results. Finally, to enhance the credibility of the ceRNA network, we validated the correlation between qualified mRNAs, miRNAs, and lncRNAs through Pearson correlation analysis and visualized through R software.

### Statistical analysis

All the statistical analyses were conducted through R software and bioinformatic tools mentioned above. The two-tailed Student’s t-test was applied to analyze the relative expression levels of miRNA. Correlations between RNA expression were evaluated through Pearson correlation analysis. A *p* value < 0.05 was set as a statistical difference.

## Results

### DEGs identification

**Fig. 1:**
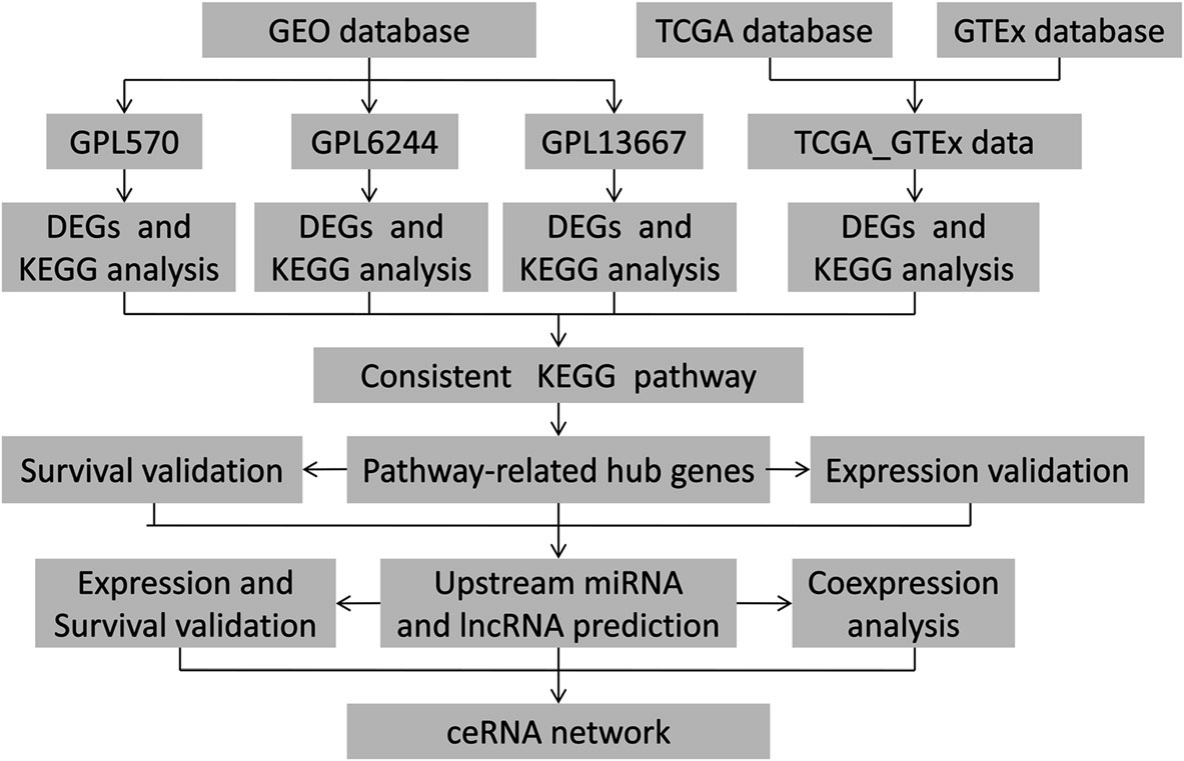
Workflow presenting the process of establishing the pathway-related ceRNA network in pancreatic cancer

**Fig. 2:**
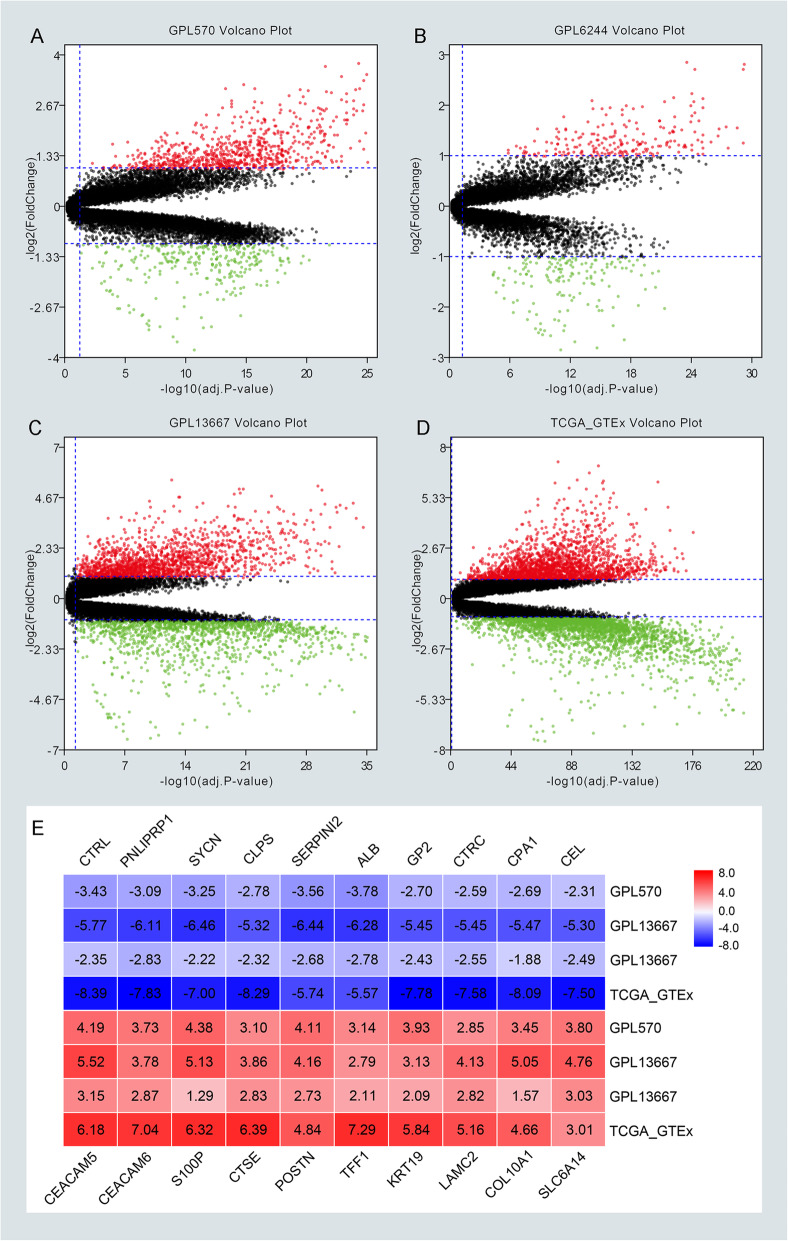
Screening differentially expressed genes (DEGs) among four groups by R software (v3.6.1). **a**–**d** The volcano plots of DEGs in GPL570, GPL6244, GPL13667, TCGA and GTEx with thresholds of |log2FC| > 1, adjust *P* value < 0.05. The red dots and green dots represent the upregulated and downregulated DEGs separately. The black dots mean no significantly different genes. **e** The top 10 up and down regulated DEGs identified by an integrated analysis of four groups

The workflow of our study was depicted in Fig. 1. Briefly, eight GEO datasets were divided into three groups, including 110 PC samples and 67 normal samples in GPL570, 120 PC samples, and 112 normal samples in GPL6244, 118 PC tissues, and 13 normal tissues in GPL13667. TCGA and GTEx database of PC were enrolled in 178 PC tissues and 171 normal tissues. The detail of each group can be found in Supplementary Table 1 (Table S1). Next, DEGs in each group were identified and displayed in the volcano plot with thresholds of |log2FC| > 1 and adjust *P* value < 0.05 (Fig. 2a–d). As depicted in Venn plots (Supplementary Figure 1A–B), we integrated commonly expressed genes that were intersected in each group, and successfully identified 170 up-regulated and 99 down-regulated DEGs. The top 10 up and down regulated DEGs identified by integrated analysis of all groups are shown in Fig. 2e. Also, We provided the result of DEGs among four groups and common DEGs in supplementary Table 4 (Table S4), and DEGs in each group were chosen for the following enrichment analysis.

### Enrichment analysis for DEGs

**Fig. 3:**
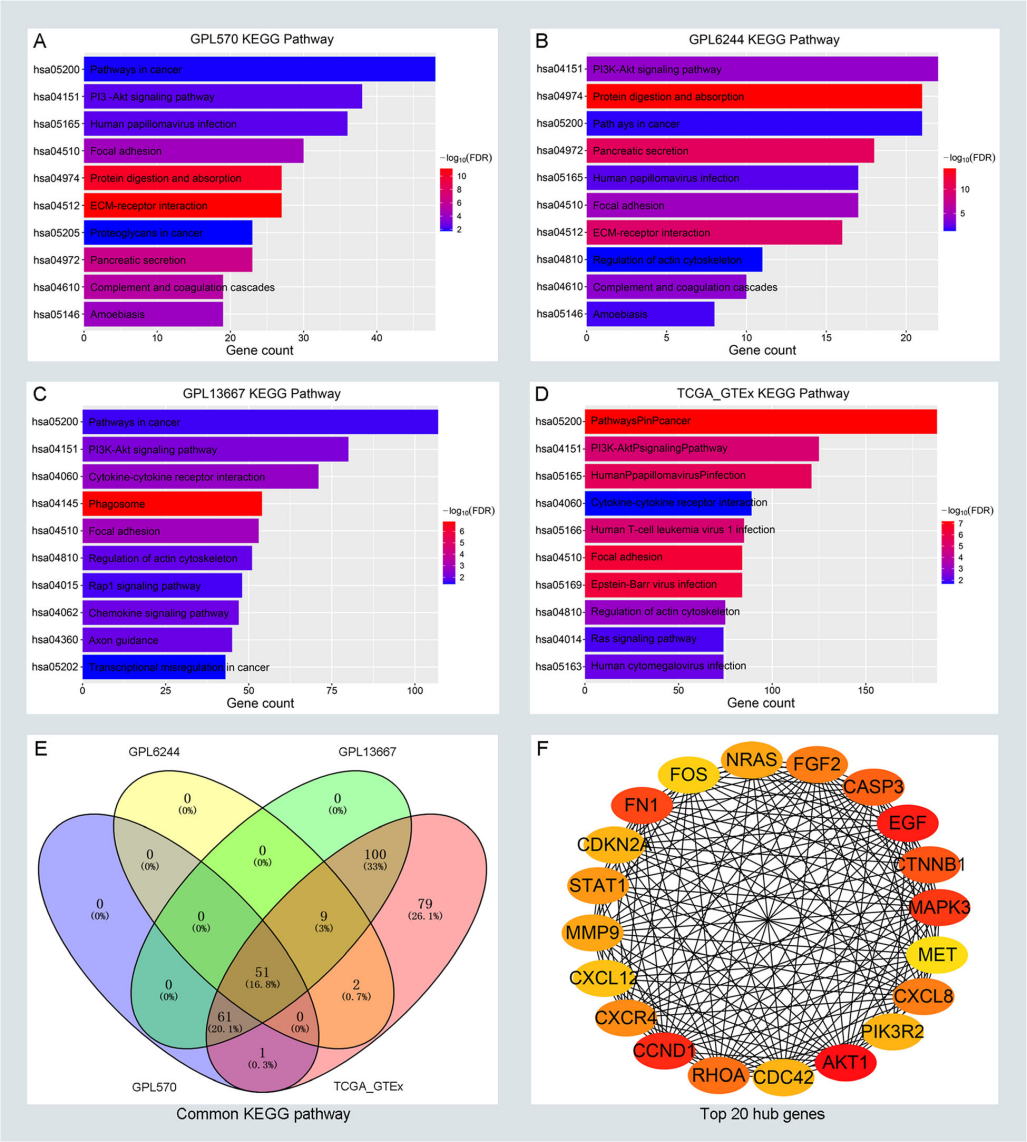
KEGG enrichment analysis for DEGs and identifying hub genes in cancer-related pathways of PC. The top ten significantly enriched the KEGG pathway in GPL570 (**a**), GPL6244 (**b**), GPL13667 (**c**), and TCGA_GTEx (**d**) respectively. **e** The distribution of enriched KEGG pathways in GPL570, GPL6244, GPL13667, and TCGA_GTEx. **f** The top 20 hub genes of DEGs in pathway in cancer. The depth of color represents the connection degree of genes. Red represents a high connection degree and yellow represents low connection. The number of lines represents the degree of connection between genes, and the more lines, the tighter the connection

To detect the potential biological functions among DEGs in each group, GO enrichment analysis and KEGG pathway analysis was conducted. The result of the GO enrichment analysis was shown in Supplementary Figure 2A–D. It is well acknowledged that cancer-related pathways play key roles in the progression of PC, and genes with similar functions cluster together to form a regulatory pathway. So we try to figure out the similarity of the KEGG pathway among DEGs. As depicted in Fig. 3a–d, the significant KEGG pathway with adjust *P* value < 0.05 were commonly enriched in the pathway in cancer, PI3K-Akt signaling pathway and Focal adhesion pathway, which indicated that those pathways were closely related to the progression of PC. There are 51 significant pathways which commonly enriched in every group (Fig. 3e). As depicted in Supplementary Figure 3A–D, most of the DEGs were enriched in the pathway of pathway in cancer, which suggested that pathway in cancer played key roles in the progression of pancreatic cancer. Additionally, we focus on DEGs that enriched in the pathway of pathway in cancer and chosen for subsequent analysis.

### Screening and validation of hub genes

**Fig. 4:**
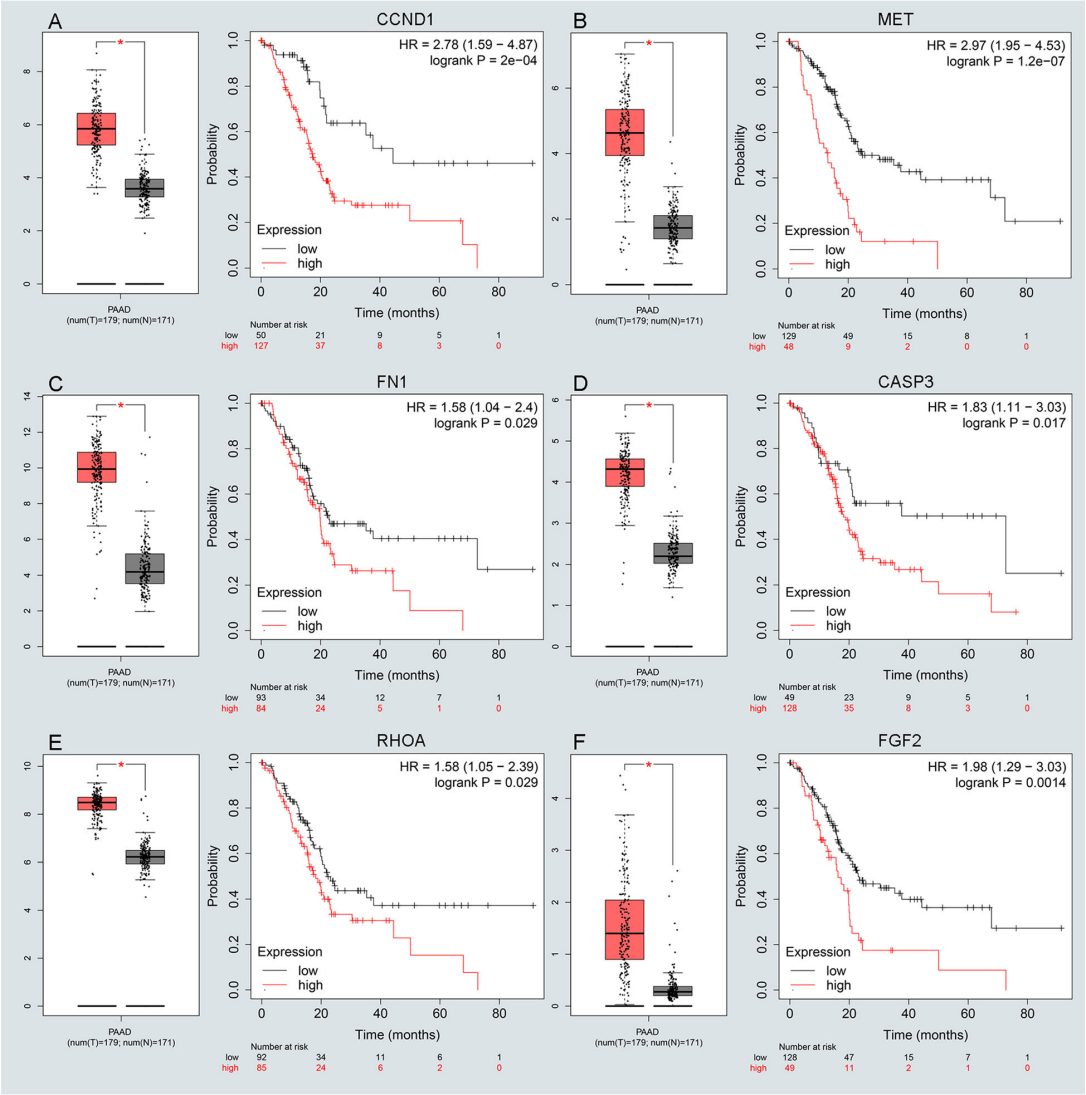
Screening and validating the expression roles and prognosis values of key genes in PC. **a**-**f** Validating expression roles and prognosis values of key genes in hub genes using GEPIA and Kaplan–Meier plotter databases

There are altogether 224 genes which enriched in the pathway of pathway in cancer. To understand the mutual interaction between DEGs and pathway in cancer, a PPI network was constructed. Also, we calculated the node degree of the PPI network using cytoHubba tools from Cytoscape software and identified the top 20 hub genes in the pathway of pathway in cancer (Fig. 3f). Subsequently, GEPIA and KM plot database was performed to assess the expression and prognosis roles of pathway-related hub genes. For pathway-related hub genes, 19 genes were up-regulated in PC and one that was down-regulated in PC. Only EGF expressed at a low level but didn’t associate with a good prognosis in PC (Supplementary Figure 4F). Notably, CCND1, FN1, and MET were up-regulated in all groups as depicted in Supplementary Figure 1D-F. There are eleven genes (CCND1, FN1, CTNNB1, CASP3, RHOA, FGF2, CXCL8, STAT1, MMP9, NRAS, and MET) that were not only significantly up-regulated in PC but also obviously related to poor prognosis of PC (Fig. 4a-f and Supplementary Figure 4A–E), and selected for candidate hub genes.

### Identification and validation of upstream miRNA

Based on the result from candidate hub genes, we further identified the upstream miRNA of those genes through the miRTarBase database that included the experimentally validated microRNA-target interactions. Only these miRNAs that proved by strong evidence, including reporter assay, western blot, and qPCR methods were considered as candidate miRNAs. There are a total of 146 miRNAs that were predicted to regulate 11 hub genes (Table S2). Next, miRNA-Seq data were obtained from TCGA databases to evaluate the expression role of candidate miRNAs, and the KM plot database was selected for verifying the prognostic value of candidate miRNAs. As shown in Supplementary Figure 5A–D, we confirmed four miRNAs related to pathway-related hub genes (hsa-miR-20b, hsa-miR-139, hsa-miR-451a, and hsa-miR-144), which were not only expressed down-regulated in PC but also linked with poor prognosis in PC. Additionally, we validated the survival outcome of four miRNAs in the OncoLnc database to increase the reliability of our results. The results indicated that four miRNAs were associated with good survival, *P* < 0.05 (Supplementary Figure 6A–D). All the qualified miRNAs were selected for further tests.

### Prediction and validation of upstream lncRNA

**Fig. 5:**
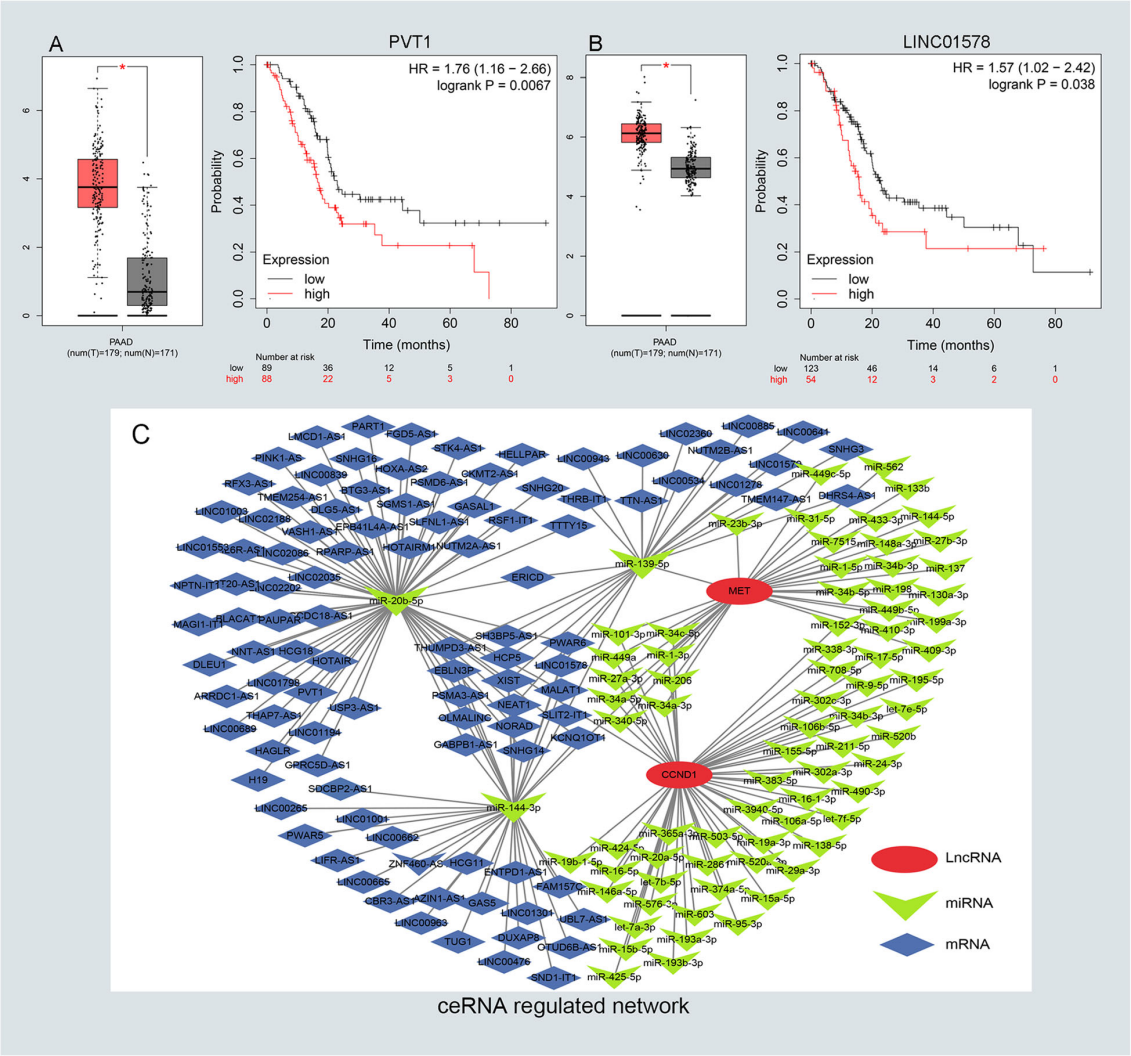
Identifying the key long noncoding RNA and constructing the pathway-related ceRNA network in PC. **a-b** Validating the expression and prognostic value of four key lncRNAs using GEPIA and Kaplan–Meier plotter databases. **c** The potential mRNA-miRNA-lncRNA regulatory network related to PC prognosis was drawn by Cytoscape software (v3.7.2). The ellipse, triangle, and diamond shape represents mRNA, miRNA, and lncRNA respectively

To predict the upstream lncRNA of candidate miRNAs, the miRNet database was employed for screening miRNA-linked lncRNAs. According to prediction in Table S3, we have found out 171 lncRNAs for four down-regulated miRNAs. Next, GEPIA and KM plot database was conducted to evaluate the expression role and prognostic value of predicted lncRNAs. According to the ceRNA hypothesis motioned above, we screened out two eligible lncRNAs (PVT1 and LINC01578) associated with down-regulated miRNAs that were both significantly up-regulated in PC and indicated dismal survival (Fig. 5a–b). Finally, we identified the eligible lncRNAs, miRNAs, and mRNAs that not only satisfied the standards of expression and prognosis but also complied with the hypothesis of ceRNA network (Fig. 5c).

### Construction and verification of ceRNA network

**Fig. 6:**
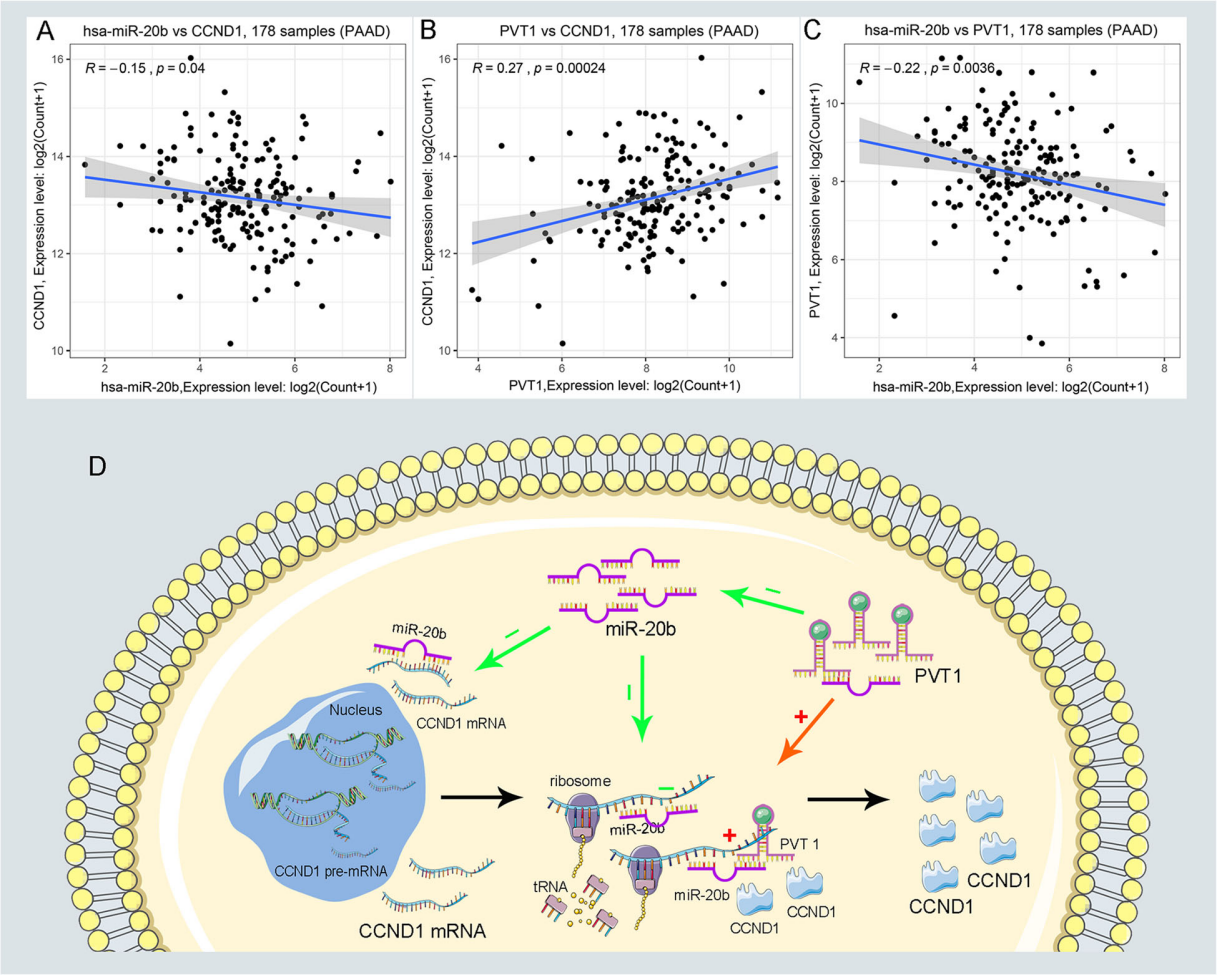
The coexpression analysis of ceRNA and schematic representations of PVT1/miR-20b/CCND1 ceRNA regulatory network. **a**-**c** The coexpression analysis indicated PVT1 was negatively correlated with miR-20b and meanwhile positively correlated with CCND1. **d** Schematic representations of pathway-related PVT1/miR-20b/CCND1 ceRNA regulatory network in the progression of PC

After previous prediction and validation, we constructed a pathway-related lncRNA-miRNA-mRNA ceRNA network. There were three ceRNA networks, including PVT1/miR-20b/CCND1, LINC01578/miR-139/MET, and LINC01578/miR-144/MET, which was constructed by our prediction. It’s widely accepted that the eligible miRNA has an opposite interaction with mRNA and lncRNA, whereas lncRNA has a positive coexpression relationship with mRNA. Furthermore, the co-expression analysis was performed to validate the inter-relationships among lncRNA-miRNA, lncRNA-miRNA, and miRNA-mRNA. Ultimately, we successfully established the PVT1/miR-20b/CCND1 cancer-related ceRNA network, which was not only significantly associated with the prognosis of PC patients but also played key roles in the progression of PC (Fig. 6a-c Supplementary Figure 7A–E). Finally, the PVT1/miR-20b/CCND1 pathway-related ceRNA network and its potential roles in the progression of PC was vividly displayed in schematic representations (Fig. 6d).

## Discussion

Although pieces of evidence have indicated that lncRNAs play regulatory roles in oncogenesis and tumor progression of various cancers, the potential mechanisms of how lncRNAs exert their regulatory roles in PC remain undefined [25]. Recently, a great deal of research has suggested that lncRNAs can interact with miRNAs and further regulate downstream mRNA expression [7]. Notably, lnRNAs, miRNAs, and mRNAs are inclined to function as a unit, not simply one to one interactions in PC. For instance, Hai Gao et al. reported that lncRNA ZEB2-AS1 was involved in encouraging tumor growth and invasion of PC through the ZEB2-AS1/miR-204/HMGB1 ceRNA network [26]; Xiong et al. indicated that GSTM3TV2 functioned as a ceRNA and negatively regulated let-7 expression, thereby leading to the PC progression and chemo-resistance through upsetting the expression of LAT2 and OLR1 [27]; Li et al. demonstrated that NORAD may function as a ceRNA to regulate the expression of the small GTP binding protein Rho A through competition for hsa-miR-125a-3p, thereby promoting EMT [28]. More importantly, it is well known that cancer-related pathways play significant roles in the progression of PC, and genes with similar functions cluster together to form a regulatory pathway [3]. However, it is unclear about the relationship between the ceRNA network and cancer-related pathways. Therefore, it’s necessary to elucidate the role of pathway-related ceRNA network in tumor origin and progress.

In the present study, we successfully identified a novel pathway-related PVT1/miR-20b/CCND1 ceRNA network involved in PC progression through Integrated analysis. Firstly, we divided eight GEO datasets into three groups according to their platform, and combined TCGA and GTEx databases as a new group. Next, we screened out DEGs and carried out KEGG enrichment analysis in each group, and looked for all the genes in the most enriched pathway. Then, those DEGs in KEGG were visualized in the PPI network and identified as hub genes according to their node degrees calculated by cytoHubba tool. The expression role and survival value of hub genes were validated using GEPIA and KM plotter databases separately. Eleven qualified genes met the criteria of expression validation and survival analyses. Notably, their oncogenic roles were also detected in PC progression. For example, there was an 8.1% actionable alterations of CCND1 in PC, which means that treatments for CCND1 could be implemented to diminish its impact on the cell cycle [29, 30]. Non-coding RNAs can function by regulating mRNA. Therefore, the potential role of the PVT1/miR-20b/CCND1 ceRNA network might closely relate to CCND1. CCND1 (Cyclin D1) is an important factor that regulates cell transition from the G1/S phase to G1 phase. Studies have shown that CCND1 plays an important role in the formation, proliferation, metastasis, and drug resistance of PC [31, 32]. So, PVT1/miR-20b/CCND1 ceRNA network might take part in the formation, proliferation, metastasis, and drug resistance of PC. The MET/HGF axis is involved in the complex crosstalk between tumor and stroma, especially in the interaction between cancer cells and activated PSCs, thereby favors the progression and metastasis of pancreatic cancer [33]. MMP-9 promoted disease progression to PDAC, while the deficiency of MMP-9 resulted in more invasive tumors and an increase in the desmoplastic stroma [34].

Additionally, the upstream miRNAs of hub genes were predicted and validated by relative databases motioned above. Four qualified miRNAs were screened out as key miRNAs. Similarly, some of the miRNAs were also involved in the development of PC. For instance, Sandhu et al. demonstrate that MiR-139-5p/SLC7A11 served as a tumor suppressor was able to inhibit invasion and metastasis of PC by direct-acting with PI3K/Akt Signaling Pathway [35]. Sureban et al. proved that miR-144 could mediate the low expression of Notch-1, which interacted with DCAMKL-1 to regulates the epithelial-mesenchymal transition of PC [36]. Zhao et al. illuminated that exosome-encapsulated miR-451a functioned as a high-sensitive liquid biomarker, which could predict the overall survival of high-risk PC patients [37]. Moreover, the upstream lncRNAs of key miRNAs were identified. But only two lncRNAs conformed to expression and prognostic standards. LncRNAs PVT1 was reported to facilitate PC proliferation and migration via sponge with miR-448, thereby regulating the expression of SERBP1 [38]. Also, You et al. demonstrate that gemcitabine therapy inhibited PVT1 expression but promote its encoded miRNAs miR-1207 level, and over-expression of miR-1207 enhanced the chemo-sensitivity of PC cells to gemcitabine [39].

Pathway in cancer is a kind of signaling pathway related to cancer, including the Notch pathway, Hedgehog pathway, Wnt/β-catenin pathway, and so on. The detailed description can be seen from https://www.kegg.jp/pathway/hsa05200. Many studies have focused on the Pathway in cancer [40, 41], PI3K-Akt signaling pathway and Focal adhesion pathway [42, 43], indicating that those pathways are closely related to PC development. Therefore, the cancer-related pathway is an important biological process, which not only carries a variety of biological functions but also is closely related to the development and occurrence of many disease processes, especially in cancer. In this work, we established and validated a pathway-related PVT1/miR-20b/CCND1 ceRNA network in the progression of PC, which perfectly satisfying all the conditions of the ceRNA hypothesis.

Although a few studies have already assessed the role of ceRNA in the progression of PC [44, 45], few studies focus on the pathway-related ceRNA axis using an Integrated analysis. Also, to our knowledge, it is the first study of PC that constructed a pathway-related ceRNA network in the progression of PC. Inevitably, some limitations can be found in our study. First, The results of our common DEGs are slightly different from Lu et al. [46]. This difference might result from our data selection. It is like the “Buckets effect” that the group with the fewest DEGs (GPL6244) determines the number of common DEGs. Also, we didn’t stratify the samples based on their clinical characteristics, such as sex. A recent study has indicated that incorporating sex as a biological variable is rewarding to better know cancer mechanisms [47]. Furthermore, we verify the expression and prognostic value using online databases rather than the date from clinical samples, which will undermine our work’s credibility. That’s the reason why we constructed the ceRNA axis through comprehensive analysis and validated the data with the same conditions. Last, we identified a ceRNA network mainly based on their expression and prognostic value, which might overlook some valuable information.

## Conclusions

In summary, by Integrated analysis and validation, we successfully constructed a pathway-related PVT1/miR-20b/CCND1 ceRNA regulatory network, in which all RNAs remarkably related to the prognosis of patients with PC. In addition to the prognostic roles of this network, it also provides some key clues for molecular mechanism explorations of PC in the future.

## Supplementary information

**Additional file 1: Figure S1.** The distribution of DEGs and hub genes in four groups. (A-B) The intersection of upregulated DEGs and downregulated DEGs in four groups, respectively. (C) The top 20 hub genes of common DEGs. (D-F) The expression of CCND1, FN1, and MET in GPL570, GPL6244, and GPL13667 databases.

**Additional file 2: Figure S2.** GO term enrichment analysis for DEGs in four groups, respectively. The top ten enriched biological processes, cellular components, and molecular function of DEGs in GPL570 (A), GPL6244 (B), GPL13667 (C), and TCGA_GTEx (D).

**Additional file 3: Figure S3.** The distribution of DEGs in four groups related to the top three KEGG pathways. Top three KEGG pathways related to DEGs in GPL13667 (A), GPL6244 (B), GPL570 (C), and TCGA_GTEx (D) were drawn by Cytoscape software (v3.7.2). The octagon represents DEGs, and the depth of the octagon represents the value of adj. *P*-value. The circle means enriched KEGG pathway, and the size of the circle represents how many genes are enriched in the KEGG pathway.

**Additional file 4: Figure S4.** Screening and validating the expression roles and prognostic values of key genes in PC. (A - F) Validating expression roles and prognosis values of key genes in hub genes using GEPIA and Kaplan–Meier plotter databases.

**Additional file 5: Figure S5.** Screening and validating the expression roles and prognostic values of key miRNAs in PC. (A - D) Validating the expression roles in TCGA databases, and prognosis values of key miRNAs using Kaplan–Meier plotter databases.

**Additional file 6: Figure S6.** Validating the survival outcome of key miRNAs in PC. (A - D) Validating prognosis values of key miRNAs using OncoLnc database.

**Additional file 7: Figure S7.** Identifying pathway-related ceRNA regulated network through correlation analysis. Only PVT1/ miR-20b/CCND1 ceRNA axis met the correlation analysis, and other ceRNA networks failed the criteria that lncRNAs positively associated with mRNAs while miRNAs negatively related to lncRNAs and mRNAs (A - E).

**Additional file 8: Table S1.** Details of eight GEO datasets, TCGA, and GTEx databases included in this study.

**Additional file 9: Table S2.** The mRNA-miRNA pairs predicted by the miRTarBase database.

**Additional file 10: Table S3.** The miRNA-lncRNA pairs predicted by the miRNet database.

**Additional file 11: Table S4.** Differentially expressed genes among four groups and common DEGs.

## Data Availability

All data generated or analyzed during this study are included in this published article and its supplementary information files.

## Abbreviations

TCGA: The Cancer Genome Atlas
GTEx: Genotype-Tissue Expression
ceRNA: Competing endogenous RNA
DEGs: Differentially expressed genes
PC: Pancreatic cancer
GEO: Gene Expression Omnibus
GO: Gene Ontology
KEGG: Kyoto Encyclopedia of Genes and Genomes
