# Supplementary figures and images for: Integrated analysis identifies a pathway-related competing endogenous RNA network in the progression of pancreatic cancer
Source: BMC Cancer. 2020 Oct 2;20:958. doi: 10.1186/s12885-020-07470-4 (PMC7532576; doi:10.1186/s12885-020-07470-4)

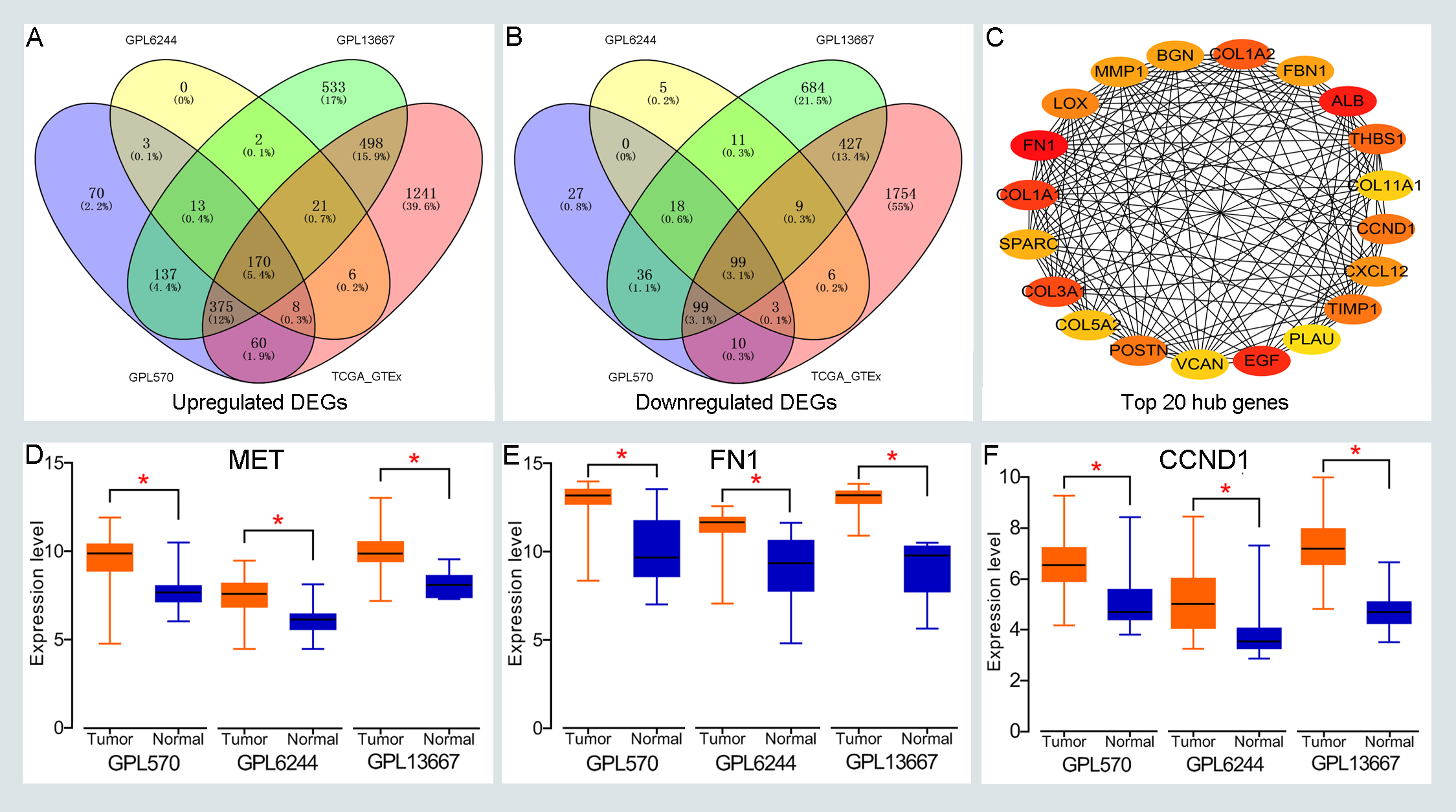

Supplement: Supplementary file 1 — Additional file 1: Figure S1. The distribution of DEGs and hub genes in four groups. (A-B) The intersection of upregulated DEGs and downregulated DEGs in four groups, respectively. (C) The top 20 hub genes of common DEGs. (D-F) The expression of CCND1, FN1, and MET in GPL570, GPL6244, and GPL13667 databases. [file 12885_2020_7470_MOESM1_ESM.tif]

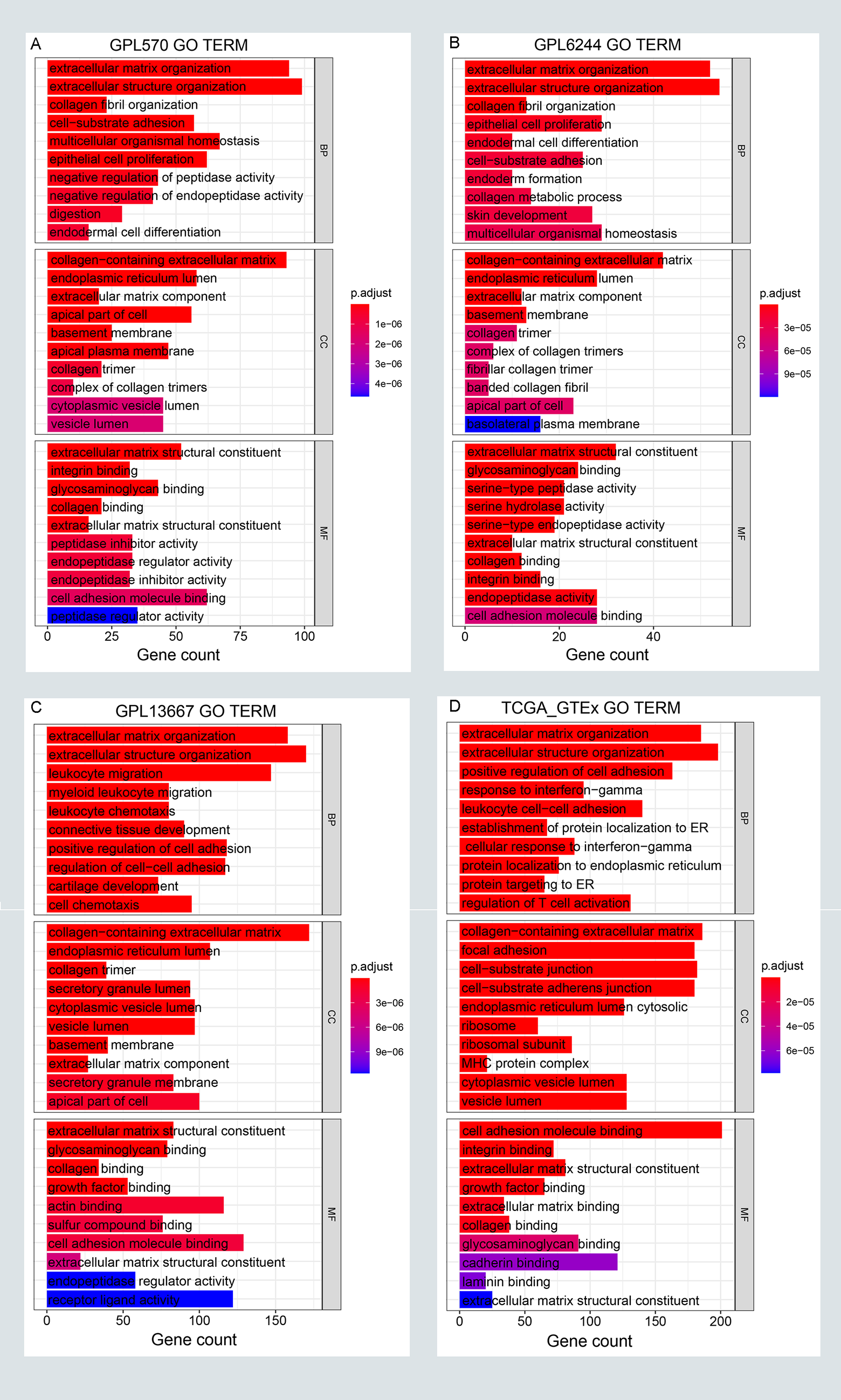

Supplement: Supplementary file 2 — Additional file 2: Figure S2. GO term enrichment analysis for DEGs in four groups, respectively. The top ten enriched biological processes, cellular components, and molecular function of DEGs in GPL570 (A), GPL6244 (B), GPL13667 (C), and TCGA_GTEx (D). [file 12885_2020_7470_MOESM2_ESM.tif]

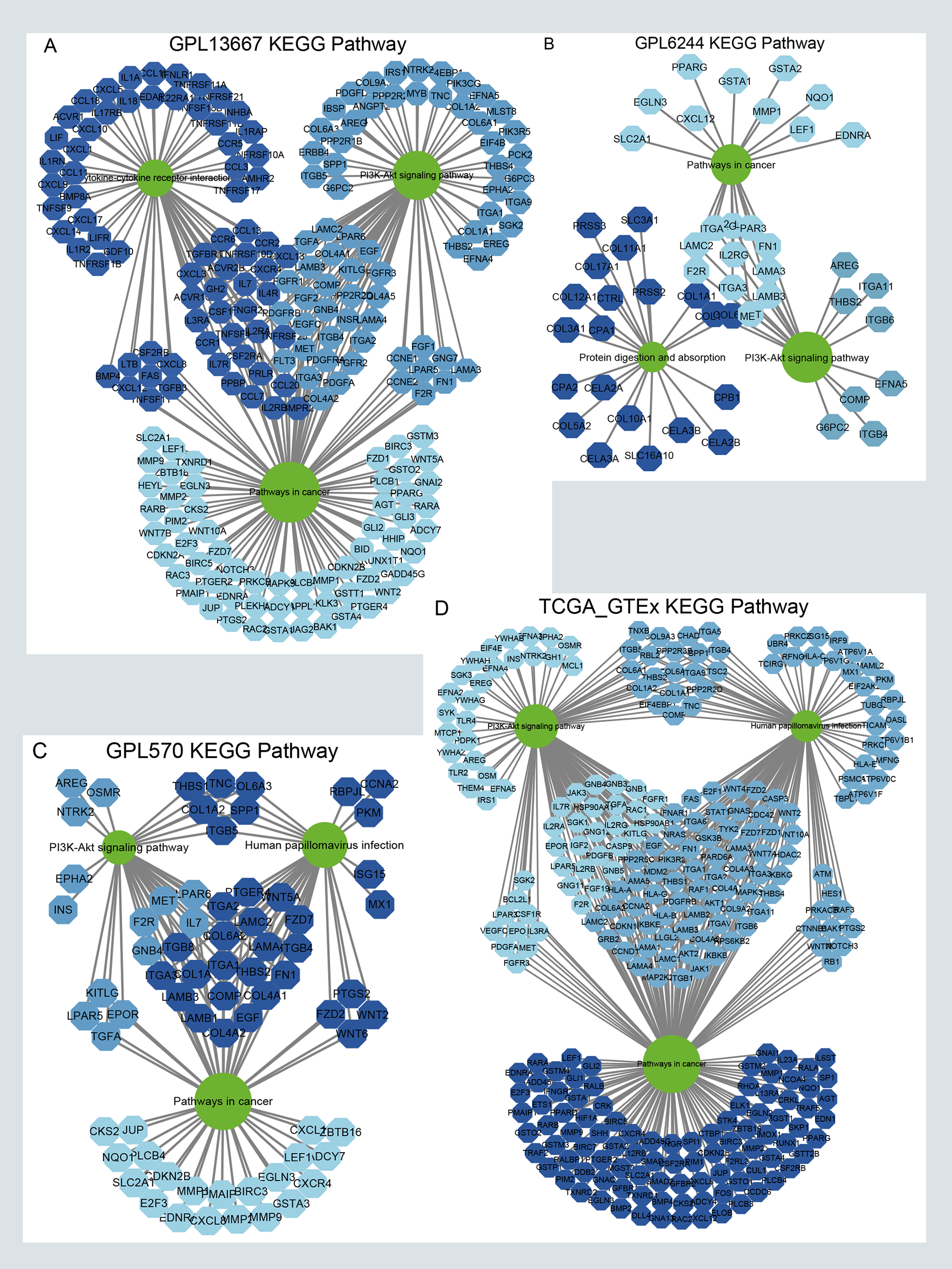

Supplement: Supplementary file 3 — Additional file 3: Figure S3. The distribution of DEGs in four groups related to the top three KEGG pathways. Top three KEGG pathways related to DEGs in GPL13667 (A), GPL6244 (B), GPL570 (C), and TCGA_GTEx (D) were drawn by Cytoscape software (v3.7.2). The octagon represents DEGs, and the depth of the octagon represents the value of adj. P-value. The circle means enriched KEGG pathway, and the size of the circle represents how many genes are enriched in the KEGG pathway. [file 12885_2020_7470_MOESM3_ESM.tif]

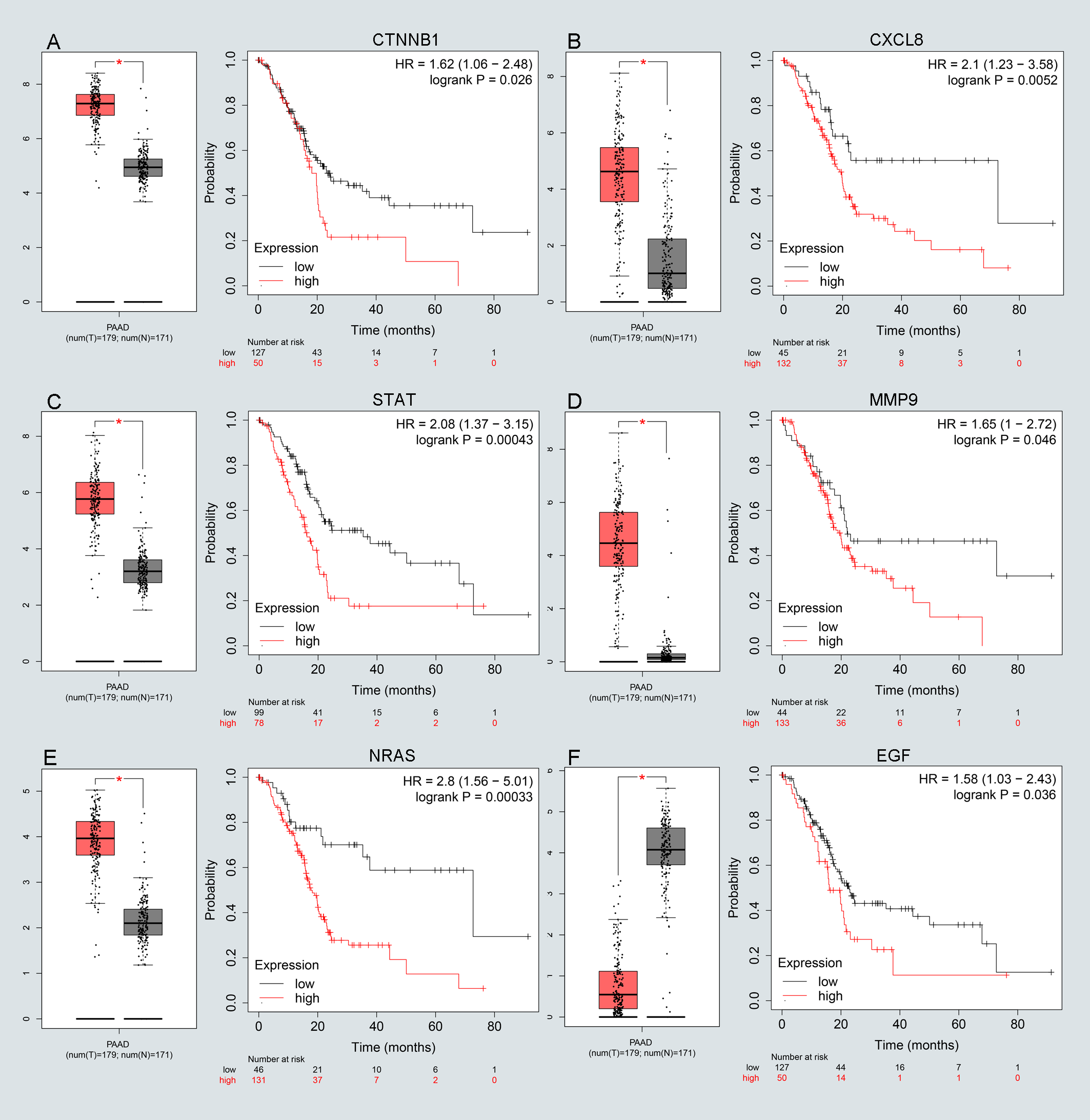

Supplement: Supplementary file 4 — Additional file 4: Figure S4. Screening and validating the expression roles and prognostic values of key genes in PC. (A - F) Validating expression roles and prognosis values of key genes in hub genes using GEPIA and Kaplan–Meier plotter databases. [file 12885_2020_7470_MOESM4_ESM.tif]

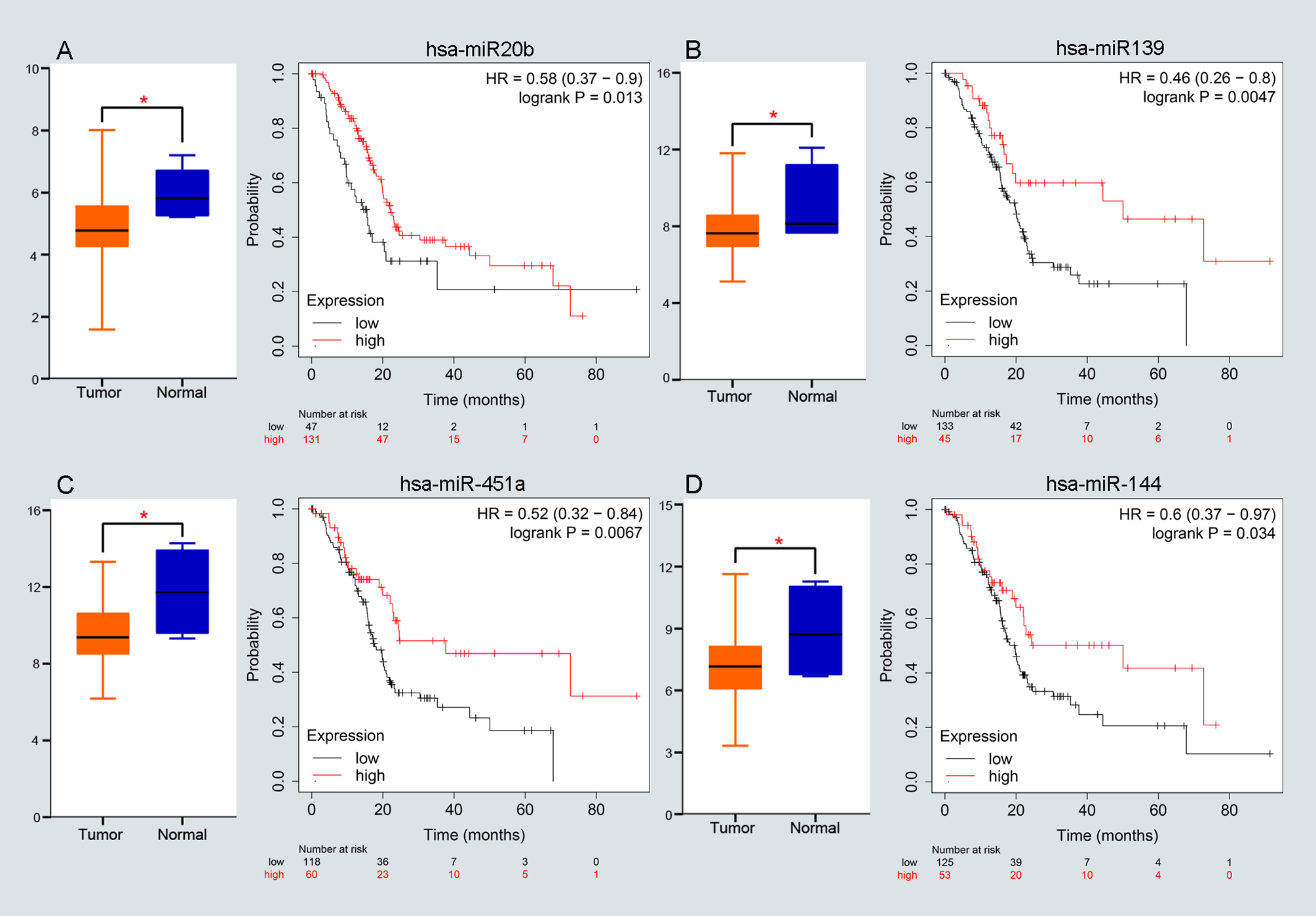

Supplement: Supplementary file 5 — Additional file 5: Figure S5. Screening and validating the expression roles and prognostic values of key miRNAs in PC. (A - D) Validating the expression roles in TCGA databases, and prognosis values of key miRNAs using Kaplan–Meier plotter databases. [file 12885_2020_7470_MOESM5_ESM.tif]

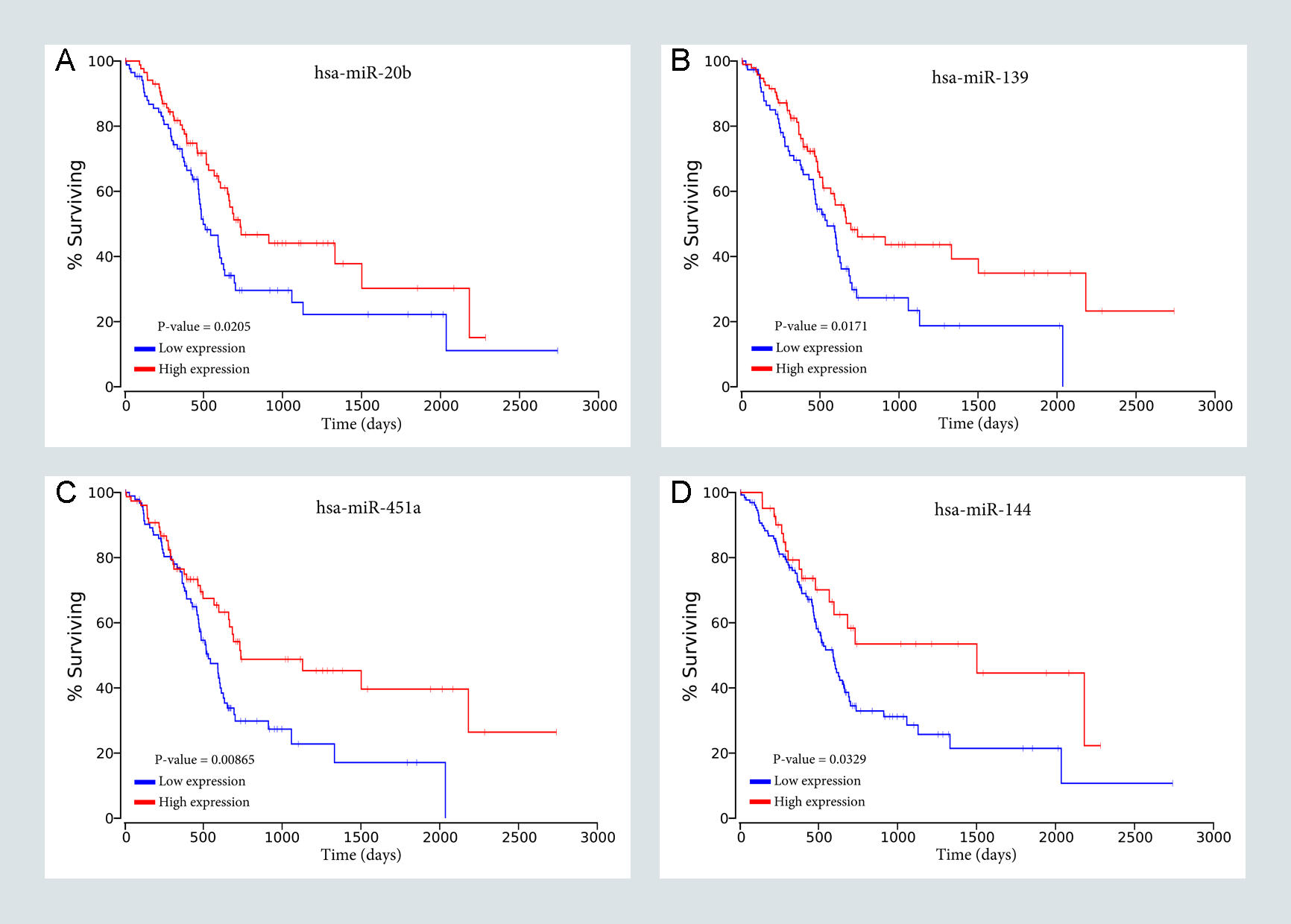

Supplement: Supplementary file 6 — Additional file 6: Figure S6. Validating the survival outcome of key miRNAs in PC. (A - D) Validating prognosis values of key miRNAs using OncoLnc database. [file 12885_2020_7470_MOESM6_ESM.tif]

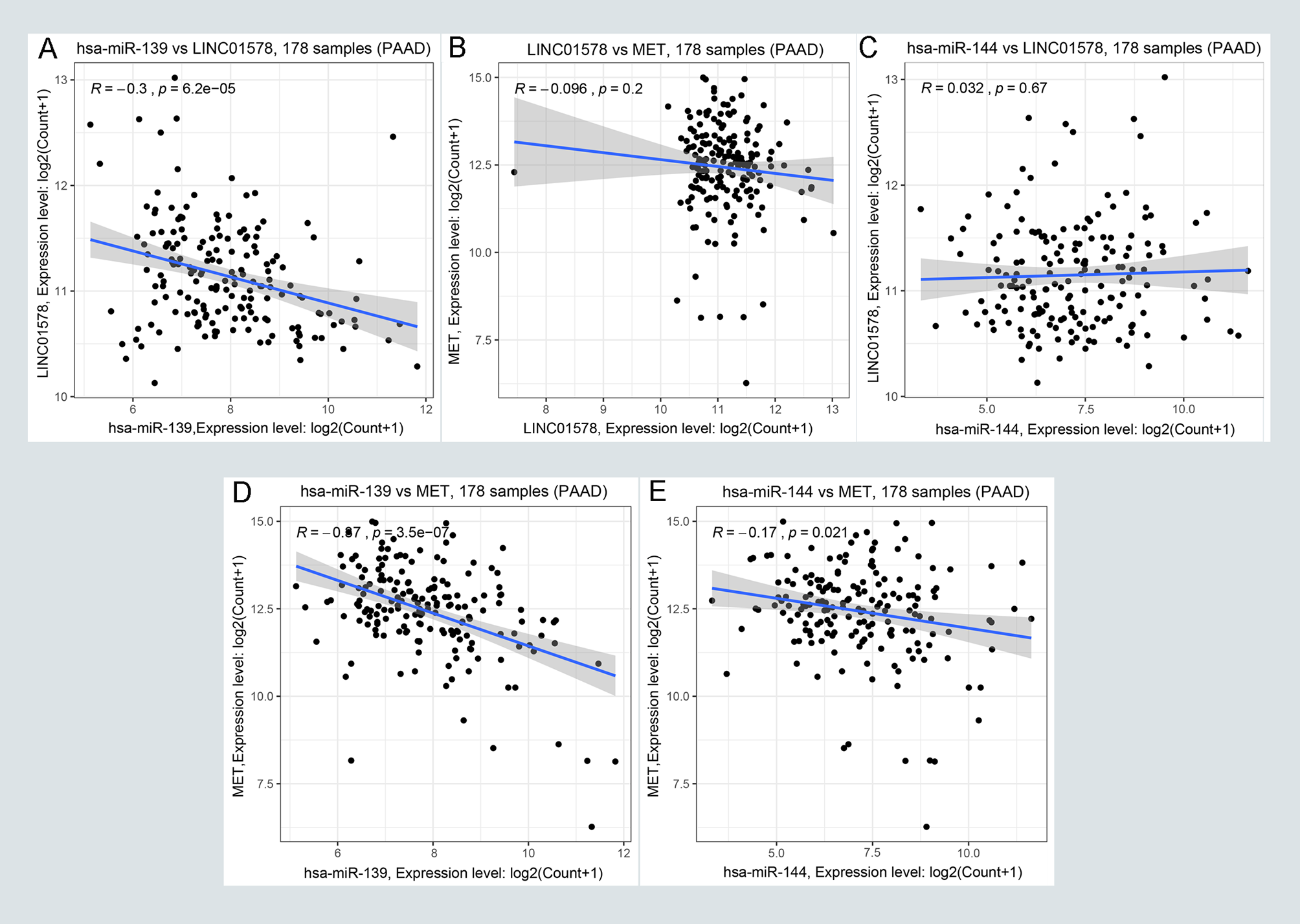

Supplement: Supplementary file 7 — Additional file 7: Figure S7. Identifying pathway-related ceRNA regulated network through correlation analysis. Only PVT1/ miR-20b/CCND1 ceRNA axis met the correlation analysis, and other ceRNA networks failed the criteria that lncRNAs positively associated with mRNAs while miRNAs negatively related to lncRNAs and mRNAs (A - E). [file 12885_2020_7470_MOESM7_ESM.tif]
