# Supplementary material for: Integrated analysis identifies a pathway-related competing endogenous RNA network in the progression of pancreatic cancer
Source: BMC Cancer. 2020 Oct 2;20:958. doi: 10.1186/s12885-020-07470-4 (PMC7532576; doi:10.1186/s12885-020-07470-4)
Supplement: Supplementary file 8 — Additional file 8: Table S1. Details of eight GEO datasets, TCGA, and GTEx databases included in this study. [file 12885_2020_7470_MOESM8_ESM.docx]

Table S1: Details of eight GEO datasets, TCGA and GTEx databases included in this study.

| Platform | Datasets | Sample size | | | Differentially expressed genes | | |
| --- | --- | --- | --- | --- | --- | --- | --- |
|  |  | Total | Tumor | Normal | Total | UP-DEGs | Down-DEGs |
| GPL570 | GSE15471 | 72 | 36 | 36 | 1128 | 836 | 292 |
|  | GSE16515 | 52 | 36 | 16 |  |  |  |
|  | GSE32676 | 32 | 25 | 7 |  |  |  |
|  | GSE71989 | 21 | 13 | 8 |  |  |  |
| GPL6244 | GSE28735 | 90 | 45 | 45 | 374 | 223 | 151 |
|  | GSE41368 | 12 | 6 | 6 |  |  |  |
|  | GSE62452 | 130 | 69 | 61 |  |  |  |
| GPL13667 | GSE62165 | 131 | 118 | 13 | 3096 | 1749 | 1347 |
| TCGA_GTEx | TCGA | 182 | 178 | 4 | 4786 | 2379 | 2407 |
|  | GTEx | 167 | 0 | 167 |  |  |  |

DEGs: Differentially expressed genes with thresholds of |log2FC| >1 and adjust P value < 0.05
