# Supplementary material for: Integrated analysis identifies a pathway-related competing endogenous RNA network in the progression of pancreatic cancer
Source: BMC Cancer. 2020 Oct 2;20:958. doi: 10.1186/s12885-020-07470-4 (PMC7532576; doi:10.1186/s12885-020-07470-4)
Supplement: Supplementary file 9 — Additional file 9: Table S2. The mRNA-miRNA pairs predicted by the miRTarBase database. [file 12885_2020_7470_MOESM9_ESM.docx]

Table S2. The mRNA-miRNA pairs predicted by the miRTarBase database.

| mRNA | miRNA |
| --- | --- |
| CCND1 | hsa-miR-20a-5p |
| CND1 | hsa-miR-195-5p |
| CCND1 | hsa-miR-193b-3p |
| CCND1 | hsa-miR-424-5p |
| CCND1 | hsa-miR-16-1-3p |
| CCND1 | hsa-miR-34a-5p |
| CCND1 | hsa-miR-503-5p |
| CCND1 | hsa-miR-16-5p |
| CCND1 | hsa-miR-15a-5p |
| CCND1 | hsa-miR-19a-3p |
| CCND1 | hsa-let-7b-5p |
| CCND1 | hsa-miR-302a-3p |
| CCND1 | hsa-miR-15b-5p |
| CCND1 | hsa-miR-449a |
| CCND1 | hsa-miR-17-5p |
| CCND1 | hsa-miR-302c-3p |
| CCND1 | hsa-miR-106b-5p |
| CCND1 | hsa-let-7e-5p |
| CCND1 | hsa-miR-365a-3p |
| CCND1 | hsa-miR-19b-1-5p |
| CCND1 | hsa-miR-520b |
| CCND1 | hsa-miR-34b-3p |
| CCND1 | hsa-miR-338-3p |
| CCND1 | hsa-miR-9-5p |
| CCND1 | hsa-miR-425-5p |
| CCND1 | hsa-miR-155-5p |
| CCND1 | hsa-miR-1-3p |
| CCND1 | hsa-let-7f-5p |
| CCND1 | hsa-miR-138-5p |
| CCND1 | hsa-miR-106a-5p |
| CCND1 | hsa-miR-27a-3p |
| CCND1 | hsa-miR-383-5p |
| CCND1 | hsa-miR-490-3p |
| CCND1 | hsa-miR-603 |
| CCND1 | hsa-miR-340-5p |
| CCND1 | hsa-miR-374a-5p |
| CCND1 | hsa-miR-193a-3p |
| CCND1 | hsa-miR-520a-3p |
| CCND1 | hsa-miR-20b-5p |
| CCND1 | hsa-miR-708-5p |
| CCND1 | hsa-miR-29a-3p |
| CCND1 | hsa-miR-24-3p |
| CCND1 | hsa-miR-152-3p |
| CCND1 | hsa-miR-206 |
| CCND1 | hsa-miR-95-3p |
| CCND1 | hsa-miR-2861 |
| CCND1 | hsa-miR-34a-3p |
| CCND1 | hsa-miR-3940-5p |
| CCND1 | hsa-miR-34c-5p |
| CCND1 | hsa-miR-101-3p |
| CCND1 | hsa-miR-146a-5p |
| CCND1 | hsa-miR-576-3p |
| CCND1 | hsa-miR-211-5p |
| CCND1 | hsa-let-7a-3p |
| FN1 | hsa-miR-200c-3p |
| FN1 | hsa-miR-200b-3p |
| FN1 | hsa-miR-1-3p |
| FN1 | hsa-let-7g-5p |
| FN1 | hsa-miR-140-3p |
| CTNNB1 | hsa-miR-200a-3p |
| CTNNB1 | hsa-miR-1826 |
| CTNNB1 | hsa-miR-214-3p |
| CTNNB1 | hsa-miR-34a-3p |
| CTNNB1 | hsa-miR-34b-5p |
| CTNNB1 | hsa-miR-34c-3p |
| CTNNB1 | hsa-miR-370-3p |
| CTNNB1 | hsa-miR-375 |
| CTNNB1 | hsa-miR-3162-3p |
| CTNNB1 | hsa-miR-150-3p |
| CTNNB1 | hsa-miR-181a-5p |
| CTNNB1 | hsa-miR-320a |
| CTNNB1 | hsa-miR-200a-5p |
| CTNNB1 | hsa-miR-506-5p |
| CTNNB1 | hsa-miR-101-3p |
| CTNNB1 | hsa-miR-885-5p |
| CASP3 | hsa-miR-98-5p |
| CASP3 | hsa-let-7a-5p |
| CASP3 | hsa-miR-30d-5p |
| CASP3 | hsa-miR-138-5p |
| CASP3 | hsa-miR-375 |
| CASP3 | hsa-miR-421 |
| CASP3 | hsa-let-7g-5p |
| CASP3 | hsa-let-7b-3p |
| CASP3 | hsa-miR-30c-5p |
| CASP3 | hsa-miR-582-5p |
| CASP3 | hsa-miR-363-3p |
| CASP3 | hsa-miR-885-5p |
| CASP3 | hsa-miR-30e-5p |
| CASP3 | hsa-miR-224-5p |
| CASP3 | hsa-miR-26a-1-3p |
| CASP3 | hsa-miR-34a-5p |
| CASP3 | hsa-miR-155-5p |
| CASP3 | hsa-let-7c-5p |
| RHOA | hsa-miR-31-5p |
| RHOA | hsa-miR-122-5p |
| RHOA | hsa-miR-155-5p |
| RHOA | hsa-miR-185-5p |
| RHOA | hsa-miR-31-3p |
| RHOA | hsa-miR-200c-3p |
| RHOA | hsa-miR-375 |
| RHOA | hsa-miR-133b |
| RHOA | hsa-miR-125a-3p |
| RHOA | hsa-miR-340-5p |
| RHOA | hsa-miR-200b-3p |
| RHOA | hsa-miR-146a-5p |
| RHOA | hsa-miR-490-3p |
| RHOA | hsa-miR-101-3p |
| RHOA | hsa-miR-133a-5p |
| RHOA | hsa-miR-200b-5p |
| RHOA | hsa-miR-483-5p |
| FGF2 | hsa-miR-16-5p |
| FGF2 | hsa-miR-503-5p |
| FGF2 | hsa-miR-186-5p |
| FGF2 | hsa-miR-195-5p |
| FGF2 | hsa-miR-424-5p |
| FGF2 | hsa-miR-152-3p |
| FGF2 | hsa-miR-196a-3p |
| FGF2 | hsa-miR-132-3p |
| FGF2 | hsa-miR-205-3p |
| FGF2 | hsa-miR-152-5p |
| FGF2 | hsa-miR-203a-3p |
| FGF2 | hsa-miR-195-3p |
| CXCL8 | hsa-miR-146a-5p |
| CXCL8 | hsa-miR-520b |
| CXCL8 | hsa-miR-155-5p |
| CXCL8 | hsa-miR-23a-3p |
| CXCL8 | hsa-miR-93-5p |
| CXCL8 | hsa-miR-203a-3p |
| CXCL8 | hsa-miR-302c-3p |
| CXCL8 | hsa-miR-302d-3p |
| CXCL8 | hsa-miR-100-3p |
| CXCL8 | hsa-miR-106a-5p |
| STAT1 | hsa-miR-145-5p |
| STAT1 | hsa-miR-146a-5p |
| STAT1 | hsa-miR-140-5p |
| STAT1 | hsa-miR-150-5p |
| STAT1 | hsa-miR-223-3p |
| STAT1 | hsa-miR-203a-3p |
| STAT1 | hsa-miR-450a-5p |
| STAT1 | hsa-miR-155-5p |
| MMP9 | hsa-miR-451a |
| MMP9 | hsa-miR-491-5p |
| MMP9 | hsa-miR-338-3p |
| MMP9 | hsa-miR-204-5p |
| MMP9 | hsa-miR-21-5p |
| MMP9 | hsa-miR-9-5p |
| MMP9 | hsa-miR-211-5p |
| MMP9 | hsa-let-7e-5p |
| MMP9 | hsa-miR-133b |
| MMP9 | hsa-miR-29b-3p |
| MMP9 | hsa-miR-9-3p |
| MMP9 | hsa-miR-524-5p |
| MMP9 | hsa-miR-302a-5p |
| MMP9 | hsa-miR-132-3p |
| MMP9 | hsa-miR-15b-5p |
| MMP9 | hsa-miR-942-3p |
| MMP9 | hsa-miR-203a-5p |
| MMP9 | hsa-miR-133a-5p |
| MMP9 | hsa-miR-143-3p |
| NRAS | hsa-let-7a-5p |
| NRAS | hsa-let-7b-5p |
| NRAS | hsa-miR-20a-5p |
| NRAS | hsa-let-7c-5p |
| NRAS | hsa-let-7b-5p |
| NRAS | hsa-miR-145-5p |
| NRAS | hsa-miR-148b-3p |
| NRAS | hsa-miR-98-5p |
| NRAS | hsa-miR-214-3p |
| NRAS | hsa-miR-26a-5p |
| NRAS | hsa-miR-181a-5p |
| NRAS | hsa-miR-98-3p |
| MET | hsa-miR-206 |
| MET | hsa-miR-34c-5p |
| MET | hsa-miR-34b-5p |
| MET | hsa-miR-34a-5p |
| MET | hsa-miR-1-3p |
| MET | hsa-miR-199a-3p |
| MET | hsa-miR-23b-3p |
| MET | hsa-miR-34b-3p |
| MET | hsa-miR-562 |
| MET | hsa-miR-340-5p |
| MET | hsa-miR-449a |
| MET | hsa-miR-449b-5p |
| MET | hsa-miR-198 |
| MET | hsa-miR-130a-3p |
| MET | hsa-miR-133b |
| MET | hsa-miR-31-5p |
| MET | hsa-miR-27a-3p |
| MET | hsa-miR-137 |
| MET | hsa-miR-410-3p |
| MET | hsa-miR-148a-3p |
| MET | hsa-miR-449c-5p |
| MET | hsa-miR-409-3p |
| MET | hsa-miR-101-3p |
| MET | hsa-miR-144-3p |
| MET | hsa-miR-7515 |
| MET | hsa-miR-433-3p |
| MET | hsa-miR-139-5p |
| MET | hsa-miR-27b-3p |
| MET | hsa-miR-34a-3p |
| MET | hsa-miR-1-5p |
| MET | hsa-miR-144-5p |
