# Supplementary material for: Integrated analysis identifies a pathway-related competing endogenous RNA network in the progression of pancreatic cancer
Source: BMC Cancer. 2020 Oct 2;20:958. doi: 10.1186/s12885-020-07470-4 (PMC7532576; doi:10.1186/s12885-020-07470-4)
Supplement: Supplementary file 10 — Additional file 10: Table S3. The miRNA-lncRNA pairs predicted by the miRNet database. [file 12885_2020_7470_MOESM10_ESM.docx]

Table S3. The miRNA-lncRNA pairs predicted by the miRNet database.

| miRNA | lncRNA |
| --- | --- |
| hsa-mir-20b-5p | SNHG14 |
| hsa-mir-20b-5p | SNHG16 |
| hsa-mir-20b-5p | SNHG20 |
| hsa-mir-20b-5p | ST20-AS1 |
| hsa-mir-20b-5p | STK4-AS1 |
| hsa-mir-20b-5p | THAP7-AS1 |
| hsa-mir-20b-5p | TMEM254-AS1 |
| hsa-mir-20b-5p | TTTY15 |
| hsa-mir-20b-5p | USP3-AS1 |
| hsa-mir-20b-5p | VASH1-AS1 |
| hsa-mir-20b-5p | XIST |
| hsa-mir-20b-5p | PART1 |
| hsa-mir-20b-5p | PAUPAR |
| hsa-mir-20b-5p | PINK1-AS |
| hsa-mir-20b-5p | PSMA3-AS1 |
| hsa-mir-20b-5p | PSMD6-AS1 |
| hsa-mir-20b-5p | PVT1 |
| hsa-mir-20b-5p | PWAR6 |
| hsa-mir-20b-5p | RFX3-AS1 |
| hsa-mir-20b-5p | RPARP-AS1 |
| hsa-mir-20b-5p | RSF1-IT1 |
| hsa-mir-20b-5p | SGMS1-AS1 |
| hsa-mir-20b-5p | SLFNL1-AS1 |
| hsa-mir-20b-5p | SLX1A-SULT1A3 |
| hsa-mir-20b-5p | SLX1B-SULT1A4 |
| hsa-mir-20b-5p | LINC01194 |
| hsa-mir-20b-5p | LINC01553 |
| hsa-mir-20b-5p | LINC01798 |
| hsa-mir-20b-5p | LINC02035 |
| hsa-mir-20b-5p | LINC02086 |
| hsa-mir-20b-5p | LINC02188 |
| hsa-mir-20b-5p | LINC02202 |
| hsa-mir-20b-5p | LMCD1-AS1 |
| hsa-mir-20b-5p | MAGI1-IT1 |
| hsa-mir-20b-5p | MALAT1 |
| hsa-mir-20b-5p | MIR181A1HG |
| hsa-mir-20b-5p | MIRLET7BHG |
| hsa-mir-20b-5p | NEAT1 |
| hsa-mir-20b-5p | NNT-AS1 |
| hsa-mir-20b-5p | NORAD |
| hsa-mir-20b-5p | NPTN-IT1 |
| hsa-mir-20b-5p | NUTM2A-AS1 |
| hsa-mir-20b-5p | OLMALINC |
| hsa-mir-20b-5p | EBLN3P |
| hsa-mir-20b-5p | EPB41L4A-AS1 |
| hsa-mir-20b-5p | ERICD |
| hsa-mir-20b-5p | FGD5-AS1 |
| hsa-mir-20b-5p | GABPB1-AS1 |
| hsa-mir-20b-5p | GASAL1 |
| hsa-mir-20b-5p | GPRC5D-AS1 |
| hsa-mir-20b-5p | H19 |
| hsa-mir-20b-5p | HAGLR |
| hsa-mir-20b-5p | HCG18 |
| hsa-mir-20b-5p | HCP5 |
| hsa-mir-20b-5p | HELLPAR |
| hsa-mir-20b-5p | HOTAIR |
| hsa-mir-20b-5p | HOTAIRM1 |
| hsa-mir-20b-5p | HOXA-AS2 |
| hsa-mir-20b-5p | IL6R-AS1 |
| hsa-mir-20b-5p | KCNQ1OT1 |
| hsa-mir-20b-5p | LINC00689 |
| hsa-mir-20b-5p | LINC00839 |
| hsa-mir-20b-5p | LINC01003 |
| hsa-mir-20b-5p | KPNA2P3 |
| hsa-mir-20b-5p | ARRDC1-AS1 |
| hsa-mir-20b-5p | ATP6V1B1-AS1 |
| hsa-mir-20b-5p | BLACAT1 |
| hsa-mir-20b-5p | BTG3-AS1 |
| hsa-mir-20b-5p | CCDC18-AS1 |
| hsa-mir-20b-5p | CKMT2-AS1 |
| hsa-mir-20b-5p | DLEU1 |
| hsa-mir-20b-5p | DLG5-AS1 |
| hsa-mir-139-5p | DHRS4-AS1 |
| hsa-mir-139-5p | ERICD |
| hsa-mir-139-5p | HCP5 |
| hsa-mir-139-5p | LINC00534 |
| hsa-mir-139-5p | LINC00630 |
| hsa-mir-139-5p | LINC00641 |
| hsa-mir-139-5p | LINC00885 |
| hsa-mir-139-5p | LINC00943 |
| hsa-mir-139-5p | LINC01278 |
| hsa-mir-139-5p | LINC01578 |
| hsa-mir-139-5p | LINC01579 |
| hsa-mir-139-5p | LINC02360 |
| hsa-mir-139-5p | MIR181A1HG |
| hsa-mir-139-5p | N4BP2L2-IT2 |
| hsa-mir-139-5p | NEAT1 |
| hsa-mir-139-5p | NUTM2A-AS1 |
| hsa-mir-139-5p | NUTM2B-AS1 |
| hsa-mir-139-5p | RN7SL832P |
| hsa-mir-139-5p | SH3BP5-AS1 |
| hsa-mir-139-5p | SLIT2-IT1 |
| hsa-mir-139-5p | SNHG3 |
| hsa-mir-139-5p | THRB-IT1 |
| hsa-mir-139-5p | THUMPD3-AS1 |
| hsa-mir-139-5p | TMEM147-AS1 |
| hsa-mir-139-5p | TTN-AS1 |
| hsa-mir-139-5p | XIST |
| hsa-mir-451a | KPNA2P3 |
| hsa-mir-451a | LINC00691 |
| hsa-mir-451a | LINC00886 |
| hsa-mir-451a | LINC01278 |
| hsa-mir-451a | NORAD |
| hsa-mir-451a | PSMG3-AS1 |
| hsa-mir-451a | SLC25A21-AS1 |
| hsa-mir-451a | SNHG12 |
| hsa-mir-451a | SNHG15 |
| hsa-mir-451a | SNHG17 |
| hsa-mir-451a | SOX21-AS1 |
| hsa-mir-451a | TTTY12 |
| hsa-mir-451a | ZSCAN16-AS1 |
| hsa-mir-144-3p | KPNA2P3 |
| hsa-mir-144-3p | AZIN1-AS1 |
| hsa-mir-144-3p | CBR3-AS1 |
| hsa-mir-144-3p | DUXAP8 |
| hsa-mir-144-3p | EBLN3P |
| hsa-mir-144-3p | ENTPD1-AS1 |
| hsa-mir-144-3p | FAM157C |
| hsa-mir-144-3p | GABPB1-AS1 |
| hsa-mir-144-3p | GAS5 |
| hsa-mir-144-3p | HCG11 |
| hsa-mir-144-3p | HCP5 |
| hsa-mir-144-3p | KCNQ1OT1 |
| hsa-mir-144-3p | LIFR-AS1 |
| hsa-mir-144-3p | LINC00265 |
| hsa-mir-144-3p | LINC00476 |
| hsa-mir-144-3p | LINC00662 |
| hsa-mir-144-3p | LINC00665 |
| hsa-mir-144-3p | LINC00963 |
| hsa-mir-144-3p | LINC01001 |
| hsa-mir-144-3p | LINC01301 |
| hsa-mir-144-3p | LINC01578 |
| hsa-mir-144-3p | MALAT1 |
| hsa-mir-144-3p | MIR17HG |
| hsa-mir-144-3p | MIR34AHG |
| hsa-mir-144-3p | NEAT1 |
| hsa-mir-144-3p | NORAD |
| hsa-mir-144-3p | OLMALINC |
| hsa-mir-144-3p | OTUD6B-AS1 |
| hsa-mir-144-3p | PSMA3-AS1 |
| hsa-mir-144-3p | PWAR5 |
| hsa-mir-144-3p | PWAR6 |
| hsa-mir-144-3p | SDCBP2-AS1 |
| hsa-mir-144-3p | SH3BP5-AS1 |
| hsa-mir-144-3p | SLIT2-IT1 |
| hsa-mir-144-3p | SND1-IT1 |
| hsa-mir-144-3p | SNHG14 |
| hsa-mir-144-3p | THUMPD3-AS1 |
| hsa-mir-144-3p | TUG1 |
| hsa-mir-144-3p | UBL7-AS1 |
| hsa-mir-144-3p | XIST |
| hsa-mir-144-3p | ZNF460-AS1 |
| hsa-mir-144-5p | ANKRD10-IT1 |
| hsa-mir-144-5p | DSCAM-AS1 |
| hsa-mir-144-5p | FTX |
| hsa-mir-144-5p | GAS5 |
| hsa-mir-144-5p | LINC00294 |
| hsa-mir-144-5p | LINC00667 |
| hsa-mir-144-5p | LINC00943 |
| hsa-mir-144-5p | LINC00963 |
| hsa-mir-144-5p | LINC01126 |
| hsa-mir-144-5p | LINC01278 |
| hsa-mir-144-5p | LINC01969 |
| hsa-mir-144-5p | LINC02305 |
| hsa-mir-144-5p | MAGI2-AS3 |
| hsa-mir-144-5p | MCM3AP-AS1 |
| hsa-mir-144-5p | MIR4697HG |
| hsa-mir-144-5p | NEAT1 |
| hsa-mir-144-5p | NUTM2B-AS1 |
| hsa-mir-144-5p | RBM26-AS1 |
| hsa-mir-144-5p | XIST |
